# The Two Substrate Reduction Therapies for Type 1 Gaucher Disease Are Not Equivalent. Comment on Hughes et al. Switching between Enzyme Replacement Therapies and Substrate Reduction Therapies in Patients with Gaucher Disease: Data from the Gaucher Outcome Survey (GOS). *J. Clin. Med.* 2022, *11*, 5158

**DOI:** 10.3390/jcm12093269

**Published:** 2023-05-04

**Authors:** Pramod K. Mistry, Priya S. Kishnani, Manisha Balwani, Joel M. Charrow, Judy Hull, Neal J. Weinreb, Timothy M. Cox

**Affiliations:** 1 Department of Medicine, Pediatrics, and Cellular & Molecular Physiology, Yale University School of Medicine, 20 York Street, New Haven, CT 06510, USA; 2 Division of Medical Genetics, Department of Pediatrics, Duke University Medical Center, Durham, NC 27710, USA; 3 Genetics and Genomic Sciences, Icahn School of Medicine at Mount Sinai, New York, NY 10029, USA; 4 Division of Genetics, Genomics, and Metabolism, Northwestern University Feinberg School of Medicine, Ann & Robert H Lurie Children’s Hospital of Chicago, Chicago, IL 60611, USA; 5 Gaucher Disease, US Medical Affairs, Sanofi, Cambridge, MA 02141, USA; 6 Department of Human Genetics, University of Miami Miller School of Medicine, Miami, FL 33433, USA; 7 Lysosomal Disorders Unit, Cambridge University Hospitals NHS Foundation Trust, Cambridge CB2 0QQ, UK

In their paper, Hughes et al. [1] evaluated the reasons and consequences for patients with type 1 Gaucher disease switching from intravenous enzyme replacement therapy (ERT) to oral substrate reduction therapy (SRT) and vice versa. We commend the authors’ efforts to add to the existing knowledge about the experience of switching between these two *classes* of therapy for Gaucher disease because their modes of action and pharmacology are distinct. However, it is important that clinicians are aware of the fundamental differences between the two oral SRTs. Eliglustat is an approved first-line therapy for adults with type 1 Gaucher disease who have compatible cytochrome P450 enzyme 2D6 (CYP2D6) metabolizer phenotypes (>90% of patients [2]) [3,4]. In contrast, miglustat is approved as a second-line therapy for adults with type 1 Gaucher disease unable to tolerate ERT [5,6]. Miglustat and eliglustat are neither chemically, metabolically, pharmacologically, nor biologically equivalent. Indeed, miglustat is, in effect, the “first generation” oral SRT for Gaucher disease, and its limitations and side-effect profile were the impetus for developing eliglustat [7].

Miglustat is a glucose analog, and eliglustat is a ceramide analog (Figure 1). Although both drugs inhibit glucosylceramide synthase activity, eliglustat is more potent and specific. The half maximal inhibitory concentration (IC_50_) is approximately three orders of magnitude lower for eliglustat (IC_50_~25 nM) [8] than for miglustat (IC_50_, 10–50 µM) [9]. Miglustat is excreted unchanged in the urine and, in the presence of renal failure, the dose must be adjusted according to the estimated glomerular filtration rate (eGFR) [5]. Eliglustat is metabolized by the hepatic cytochrome P450 enzymes CYP2D6 and CYP3A, and its approved use and dosing in renal impairment are dependent upon CYP2D6 genotype, concurrent use of medications that either inhibit or induce CYP2D6 and CYP3A, and the degree of hepatic dysfunction [3].

In terms of clinical toxicities, Hughes et al. mischaracterized the relative gastrointestinal tolerability of these two drugs when they wrote: “The most common adverse events associated with miglustat are diarrhea, flatulence, abdominal pain, weight loss, and tremor, whereas gastrointestinal effects are also common with eliglustat”. In fact, gastrointestinal side effects are substantially more common and more severe with miglustat than eliglustat. According to the miglustat label, 85% of patients experienced diarrhea and 65% experienced weight loss in clinical trials [5]. The label characterizes diarrhea as “the result of the inhibitory activity of miglustat on intestinal disaccharidases such as sucrase-isomaltase in the gastrointestinal tract leading to reduced absorption of dietary disaccharides in the small intestine, with a resultant osmotic diarrhea” [5]. Anti-diarrheal medications, specialized dose escalation, and dietary modifications are approaches that attempt, with inconsistent success, to offset the poor gastrointestinal tolerability of miglustat [10,11]. In the eliglustat clinical trials, 10% of patients reported diarrhea, which was mild or moderate, and 2% reported weight loss [2]; in long-term follow-up (1400 person-years), treatment-related diarrhea continued to be uncommon (4.6% of patients) and mild or moderate in nearly all cases [12]. Strategies to combat diarrhea are not needed with eliglustat treatment.

In addition, miglustat crosses the blood–brain barrier and has been detected in cerebrospinal fluid [13], whereas eliglustat does not accumulate in the brain and has not been detected in cerebrospinal fluid [7]. In clinical trials, 30% of miglustat-treated patients [5] and 1% of eliglustat-treated patients [2] reported tremors or exacerbation of existing tremors.

Thus, the miglustat label includes warnings for diarrhea, weight loss, peripheral neuropathy, and tremors or exacerbation of existing tremors [5]. The eliglustat label has no such warnings [3]. However, unlike miglustat, the eliglustat label has warnings for electrocardiographic changes and the potential for cardiac arrhythmias in patients with pre-existing cardiac disease, long QT syndrome, and concomitant use of Class IA and Class III antiarrhythmic medications [3]. According to pharmacokinetic/pharmacodynamic modeling [14], eliglustat concentrations predicted to cause even mild increases in mean PR, QRS, and QTc intervals (i.e., concentrations greater than 500 ng/mL) [3] are substantially higher than therapeutic levels and were not observed in any patients in the eliglustat clinical trials [12].

The Hughes et al. report also appears to contain a biased presentation of adverse event data that led to treatment-switching. Adverse events that resulted in a switch from eliglustat to ERT were collected (and reported in the article), but similar information for patients who switched from miglustat to ERT was not presented, even though most switches to ERT were in miglustat-treated patients.

The authors also did not mention that women wishing to become pregnant (for whom neither miglustat nor eliglustat is recommended due to a lack of data in pregnancy [3,4,5,6]) may opt for an anticipatory switch from SRT to ERT. There is abundant literature that ERT is safe during pregnancy and can protect against Gaucher-related pregnancy complications [15].

Eliglustat’s first-line indication in type 1 Gaucher disease and superior tolerability profile have led to far greater prescribing of eliglustat than miglustat. This is reflected in Figure 1 of the Hughes et al. article where, among 222 patients who switched from ERT to SRT, 149 switched to eliglustat and 73 switched to miglustat; thereafter, 119 of 149 (80%) eliglustat-treated patients remained on eliglustat and 15 of 73 (21%) miglustat-treated patients remained on miglustat. The first-line designation of eliglustat is supported by the clinical trials, in which improvements in blood parameters and visceromegaly in treatment-naive patients were similar between ERTs and eliglustat [16], but not miglustat [17]. Similarly, in ERT-SRT switch trials, “treatment with eliglustat maintained haematological and organ volume stability in patients with type 1 Gaucher disease who had reached therapeutic goals” [18], whereas for miglustat “a notable proportion of patients showed a gradual deterioration in some disease manifestations, suggesting that miglustat could maintain clinical stability, but not in all patients” [19].

When advising patients with type 1 Gaucher disease or third-party payers about options for oral therapy, it is important that the well-documented chemical, pharmacological, and pharmacodynamic distinctions between eliglustat and miglustat are accurately and clearly explained.

## Figures and Tables

**Figure 1:**
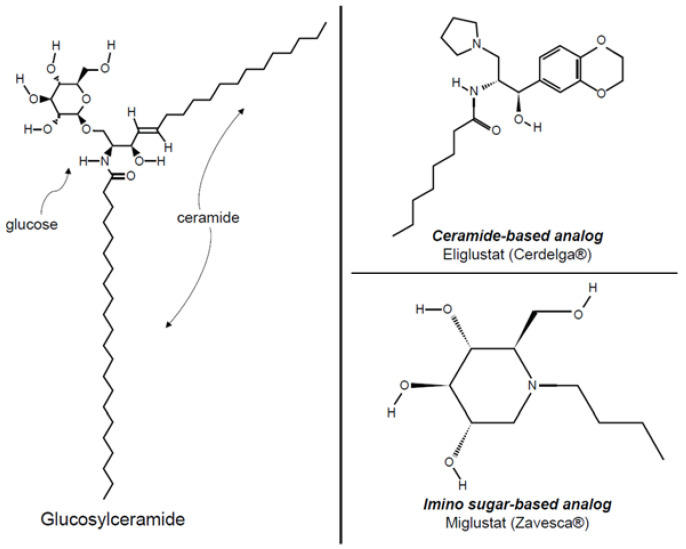
Chemical structures of glucosylceramide, eliglustat, and miglustat.
